# Distress and quality of life do not change over time in patients with operated and conservatively managed intracranial meningioma

**DOI:** 10.1007/s00701-021-05004-w

**Published:** 2021-10-13

**Authors:** Darius Kalasauskas, Naureen Keric, Salman Abu Ajaj, Leoni von Cube, Florian Ringel, Mirjam Renovanz

**Affiliations:** 1 Department of Neurosurgery, University Medical Centre, Johannes Gutenberg University of Mainz, Langenbeckstr. 1, 55131 Mainz, Germany; 2 Department of Neurosurgery, University Hospital Tübingen, Eberhard Karls University Tübingen, Tübingen, Germany; 3 Department of Neurology & Interdisciplinary Neuro-Oncology, University Hospital Tuebingen, Hertie Institute for Clinical Brain Research, Tübingen, Germany

**Keywords:** Anxiety, Depression, Distress, Meningioma, Quality of life

## Abstract

**Purpose:**

The patients’ burden with asymptomatic meningiomas and patients with good clinical outcome after meningioma resection often remains neglected. In this study, we aimed to investigate the longitudinal changes of psychological distress and quality of life in these patient groups.

**Methods:**

Patients with conservatively managed (CM) or operated (OM) meningiomas and excellent neurological status, who were screened for psychological distress during the follow-up visit (t1), were included. We performed a follow-up mail/telephone-based survey 3–6 months (t2) after t1. Distress was measured using Hospital Anxiety and Depression Scale (HADS), Distress Thermometer (DT), 36-item Short Form (SF-36), and Brief Fatigue Inventory (BFI).

**Results:**

Sixty-two patients participated in t1 and 47 in t2. The number of patients reporting increased or borderline values remained high 3 months after initial presentation, with *n* = 25 (53%) of patients reporting increased anxiety symptom severity and *n* = 29 (62%) reporting increased depressive symptom severity values. The proportion of distressed patients according to a DT score remained similar after 3 months. Forty-four percent of patients reported significant distress in OM and 33% in CM group. The most common problems among distressed patients were fatigue (t2 75%) and worries (t2 50%), followed by pain, sleep disturbances, sadness, and nervousness. Tumor progress was associated with increased depression scores (*OR* 6.3 (1.1–36.7)).

**Conclusion:**

The level of psychological distress in asymptomatic meningiomas and postoperative meningiomas with excellent outcome is high. Further investigations are needed to identify and counsel the patients at risk.

## Background

Meningiomas are common slow-growing benign lesions that originate from arachnoidal cap cells [17]. Asymptomatic meningiomas can be found in up to 2% of cranial MRIs [29]. As these lesions are usually small and do not compress surrounding structures, a common strategy recommended to the patient is wait and see. With increasing availability of cranial imaging across the globe, the number of patients diagnosed with meningioma as accidental finding is rising as well.

Despite favorable prognosis for such tumors, the patient is confronted with a diagnosis of a brain tumor. This might have severe implications on the psychological burden and the quality of life, regardless of the tumor etiology [13].

Conservatively managed (CM) patients are still required to undertake follow-up imaging, which might be stressful, there may be concerns, that the tumor will start to expand and an active treatment might be needed [13]. On the other hand, operatively managed (OM) patients can be exposed to the same kind of stressors as conservatively managed patients: they fear the tumor growth or relapse, follow-ups, or missing information about tumor behavior. The needs for psychooncological support in meningioma patients can easily be overseen, as, in contrast to gliomas, these tumors are growing more slowly, standardized questionnaires and distress screening are lacking, and physicians often fail to consider the fact that these patients might also be burdened. In our previous cross-sectional study, we found that the psychological burden in CM and OM meningioma patients is very high [12]. Even less is known, how these patients cope at home over time, as they are followed-up comparatively rarely [9]. Therefore, longitudinal assessments are required in order to observe changes over time and to assess symptoms meaningful to patients. They are also important for physicians to tailor the assessment for meningiomas patients under conservative management.

In this study, we therefore aimed to investigate the longitudinal changes of psychological distress and quality of life of conservatively managed and operated meningioma patients with excellent outcome to bridge this gap.

## Materials and methods

### Study design and patients

We conducted a single-center prospective study on patients, followed-up in a neurosurgical department at a university medical center in southwestern Germany. The patients were recruited into two groups. CM group included patients with a radiological diagnosis of intracranial meningioma and a recommendation of follow-up imaging. The patients who declined a recommended operation due to a large tumor mass, midline shift, hydrocephalus, or neurologic deficits were excluded from the study. The OM group included postoperative patients with histologically confirmed and completely resected meningioma, presenting for follow-up with an excellent outcome. The patients with postoperative neurological deficits or symptoms (except mild headache (1–2/10 on numerical analogue scale for pain), scalp hypesthesia, or forehead muscle weakness) were excluded from the study, in order to avoid bias in the assessment due to postoperative neurological deficits. No patients underwent radiation therapy or surgery before and during the study period. Other inclusion criteria were: age ≥ 18 years, agreement to participate, and no history of other tumor.

The patients who participated in the initial study [12] were asked to fill the questionnaires using post or telephone 3–6 months (t2) after the initial out-patient visit (t1). The telephone interview was always performed by the same interviewers (S.A.A. and L.C.) according to a protocol which was previously determined by the authors.

### Assessment

Patients’ performance was assessed at t1 using Eastern Cooperative Oncology Group (ECOG) performance status, and neurological status was evaluated using Neurologic Assessment in Neuro-Oncology (NANO) scale [22]. Demographic and tumor-related factors (gender, age, level of education (higher than secondary vs. other), employment, family status (living with partner), comorbidities including psychiatric treatment, tumor localization, size, growth (as described in radiological report), grade) were recorded. The tumor localization was classified into convexity, falx, anterior, middle, posterior fossa, and sella/sinus cavernosus. The time since the last significant event, i.e., tumor diagnosis for CM group, and time since operation for OM group was also recorded.

Patients’ self-assessment of health-related quality of life (HRQoL) and psychological distress was completed using SF-36, DT, HADS, and BFI questionnaires in German language at both measurements (t1 and t2).

The SF-36 [2, 32] is a validated multidimensional questionnaire measuring HRQoL. It was previously validated for the use in patients with brain tumors, including meningiomas [3]. SF-36 consists of 8 scales describing vitality (VT), physical functioning (PF), bodily pain (BP), general health perceptions (GH), physical role functioning (RP), emotional role functioning (RE), social role functioning (SF), and mental health (MH) as well as physical component summary (PCS) and mental component summary (MCS) measures [33], measured on a scale from 0 to 100. T scores, used for normalizing the scores based on normative values (mean = 50, *SD* = 10), were calculated [18]. The scales are favorably scored, meaning that higher scores indicate better health.

The Hospital Anxiety and Depression Scale (HADS) is a questionnaire measuring depressive and anxiety symptom severity, based on 14 questions [10, 35] and validated for the use in patients with brain tumors, including meningiomas [4]. The questionnaire provides 2 scores: anxiety score (HADS-A) and depression score (HADS-D) on a scale ranging from 0 to 21. For the purpose of this study, score of less than 8 was considered to be normal, 8–10 as borderline, > 10 as increased.

The Distress Thermometer (DT) is a screening questionnaire assessing psychological burden (“distress”) on a numerical analogue scale, 0–10. It is accompanied by a 34-item problem list with emotional, practical, physical, and spiritual concerns and is validated for brain tumor patients [7]. The score of ≥ 6 on DT scale was considered as significant psychological burden.

The Brief Fatigue Inventory (BFI) [20, 23] is a questionnaire assessing fatigue by 10 questions and a mean score. Eleven-step numerical rating scales are used to evaluate the severity, with higher scores indicating worse symptoms. The “worst fatigue” of ≥ 7 corresponds to clinically significant fatigue [20]. Fatigue severity on BFI scale of 0–6 was considered as “non-severe” and ≥ 7 as “severe.”

### Statistics

The sample size was estimated as 31 patients/group for the primary study, considering no difference in the HADS values between the groups as a null hypothesis, for a clinically relevant difference of ± 3; if the standard deviation is not higher than 4, the maximum possibility of type I error = 5% and that of type II error = 20%.

Categorical data were described by absolute and relative frequencies, and continuous data were described by the mean and standard deviation. Missing values of HADS, DT, and BFI questionnaires at 3 months follow-up (*n* = 61, 2.2%) were replaced using multiple imputation approach. The replacement of missing values at 0 months was not necessary as only *n* = 6 values (0.2%, all DT problem list) were missing.

The difference in the absolute values of the scores between the groups was assessed, after assessing the distribution of the tested variables by, as appropriate, paired or unpaired *t* test or Mann–Whitney *U* test or Wilcoxon signed ranks test. The difference in the distribution in categorical variables was assessed by a Chi-squared test and Fisher exact test for 2 × 2 tables. The correlation between the scores was assessed using Spearman’s rho. Logistic regression was used to evaluate the association of clinical characteristics with significant psychological burden. For regression analysis, tumor localization was further classified as falx/convexity vs. scull base, and patient age was classified as ≥ 65 years vs. younger. No correction for multiple testing was performed. Considering the multiple testings, all analyses were regarded as explorative, and *p* values were provided for descriptive reasons only. A *p* value less than 0.05 was considered statistically significant.

## Results

### Patient sample

Forty-seven patients responded to the survey at t2 (3–6 months) after initial interview. Sixty-two patients took part in the initial interview, divided equally between CM and OM groups (response rate at t2 76%).

At t2, there were 24 patients in CM and 23 in OM groups, 81% (*n* = 38) females, mean age was 61 (standard deviation, *SD* 13) years, range 37–87 years. Main patient characteristics were comparable between both time points (Table 1). The patients’ functional condition was very good, with all-except-one patient (98%) classified as ECOG 0 and 1 and mean of NANO scale 0.5 (*SD* 1.0). The most common localization of tumors was convexity (*n* = 14, 30%), followed by falx (*n* = 7, 15%). Five percent (*n* = 3) patients reported having a psychiatric disorder. Tumor growth or relapse was diagnosed in 8 (17%) cases. Only 4 (6%) patients in postoperative group had WHO grade II tumor, which made further statistical analysis concerning the influence of histological grade not possible.

**Table 1:** Main patient characteristics at the 1st and 2nd time point

	t1, 0 months	t2, 3 months
N	62	47 (76%)
Age (*SD*)	61 (13)	61 (13)
Female, %	51 (82%)	38 (81%)
Family situation, %		
Living with a partner	40 (67%)	28 (60%)
Living alone	20 (33%)	18 (38%)
Employment, %		
Full	21 (35%)	17 (36%)
Part-time	2 (3%)	2 (4%)
Unemployed	6 (10%)	5 (11%)
Retired	31 (52%)	21 (45%)
ECOG, %		
0	50 (81%)	38 (81%)
1	9 (15%)	8 (17%)
2	3 (5%)	1 (2%)
NANO scale, mean (SD)	0.4 (0.9)	0.5 (1.0)
Psychiatric disorder	3 (5%)	2 (4%)
Tumor localization, %		
Convexity	21 (36%)	14 (30%)
Falx	9 (15%)	7 (15%)
Anterior fossa	6 (10%)	5 (11%)
Middle fossa	7(12%)	7 (15%)
Posterior fossa	9 (15%)	7 (15%)
Sella/sinus cavernosus	5 (9%)	5 (11%)
WHO histological grade		(evaluated only for operated patients)
Grade I	27 (44%)	20 (43%)
Grade II	4 (6%)	3 (6%)
Time after diagnosis, months (*SD*)	*n* = 31 39 (47)	*n* = 24 45 (52)
Time after operation, months (*SD*)	*n* = 31 32 (44)	*n* = 23; 32 (47)
Tumor size, mm	24(16)	24 (17)
Tumor growth*	8 (13%)	8 (17%)

*based on radiological report, in comparison to the previous imaging study

### Psychological burden

**Fig. 1:**
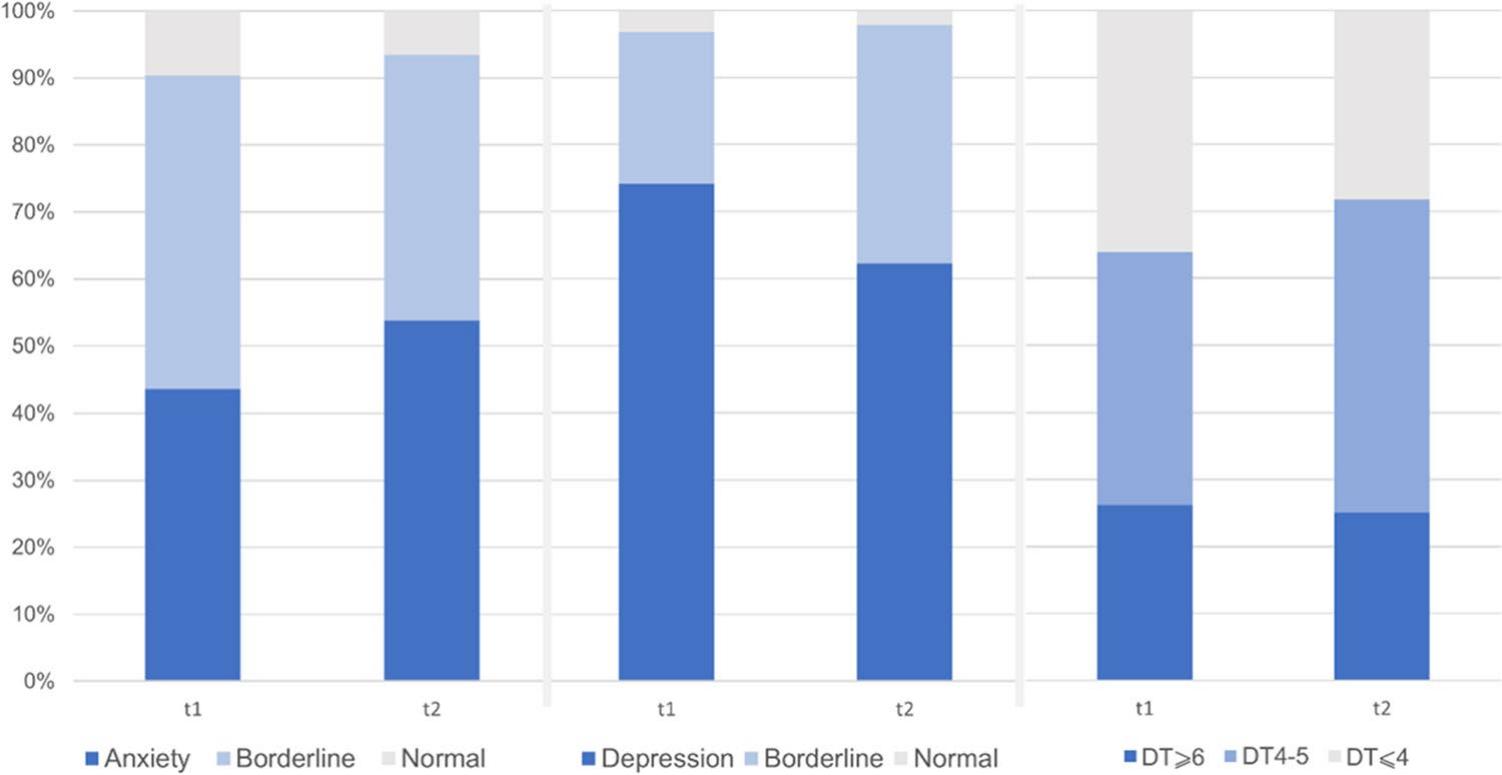
Distribution of normal, borderline, and pathological values across HADS anxiety scale (1A) and HADS depression (1B) scale as well as high, moderate, and low values on DT scale

Three months after the initial presentation, the number of patients reporting increased or borderline values remained high. In total, *n* = 25 (53%) patients reported increased anxiety symptom severity, and *n* = 29 (62%) reported increased depressive symptom severity (Fig. 1). Mean HADS-A score was 10.0 (SD 1.9) at t1 vs. 10.5 (SD 1.7) at t2, and HADS-D was 11.1 (SD 1.7) vs. 11.0 (SD 1.9), respectively. There were significantly more patients reporting increased HADS-D values in CM group at initial presentation (87% vs. 61%, *p* = 0.04, Table 2). This trend was not observed at 3 months, as the number of patients with depressive symptoms decreased significantly in CM group (*p* = 0.02). This decrease was associated with an increase in patients with borderline HADS-D values. The number of patients reporting normal values remained under 10% in all categories (Fig. 1). The proportion of patients with increased HADS-A score was similar at t1 in both groups and increased at t2 in CM group (68% vs. 39%, *p* = 0.07, Table 2).

**Table 2:** Distribution of increased values in operated and conservatively treated patients across HADS anxiety (2A), HADS depression (2B), and distress thermometer (2C) scales

		Operative	Conservative	*p* value, conservative vs. operative
HADS-A	t1	45.2%	41.9%	1.0
	t2	39.1%	68.4%	0.07
	*p* value, t1 vs. T2	0.78	0.06	
HADS-D	t1	61.3%	87.1%	0.04
	t2	65.2%	59.3%	1.0
	*p* value, t1 vs. T2	0.78	0.02	
DT	t1	36.7%	43.3%	0.79
	t2	44.3%	33.3%	0.55
	*p* value, t1 vs. T2	0.77	0.58	

The proportion of distressed patients according to a DT score remained similar after 3 months. Forty-four percent of patients reported significant distress in OM and 33% in CM group; the difference was not statistically significant (Table 2). The mean score did not change significantly in comparison to initial evaluation (4.5 (*SD* 2.5) vs. 4.8 (*SD* 2.5), *p* = 0.2). The most common problems noted on DT problem list were pain (t1 55%, t2 49%), fatigue (t1 52%, t2 49%), and worries (t1 48%, t2 39%). In patients with DT ≥ 6, the most common problems were fatigue (t1 79%, t2 75%) and worries (t1 63%, t2 50%), followed by pain, sleep disturbances, sadness, and nervousness (all 58.3% at t1 and > 65% at t2).

There were no significant changes in mental component scores (MCS) or SF-36 subscores comprising MCS in both study groups. Physical component score (PCS) was significantly lower at 3 months in CM patients (44.7 vs. 40.8, *p* = 0.03), which reflected significant differences in subscales of role limitation due to physical problems (40.8 vs. 37.4, *p* = 0.04) and bodily pain (47.8 vs. 43.1, *p* = 0.01) in CM patients. PCS and MCS scores were comparable to the values reported in the general population; however, PCS was significantly lower in CM group (47.8 vs. 40.8, *p* = 0.02). Mean GH was significantly lower at t2 in OM patient group (75.8 vs. 65.6, *p* < 0.001). Worst fatigue according to BFI was similar in both patient groups at t1 and t2 (5.6 (*SD* 2.7) vs. 5.6 (*SD* 2.9), *p* = 1.0). We found a significant correlation between MCS and DT score at 3 months (Spearman’s rho − 0.48, *p* = 0.001); however, there was no correlation between MCS and HADS-A or HADS-D.

Eight (13%) patients at t1 and 5 (8%) patients at t2 were identified who scored increased HADS-D, HADS-A, and DT scores simultaneously. However, no risk factors could be identified, most probably due to a low number of patients. Furthermore, we identified the individuals with a significant change in DT, HADS-A, and HADS-D scores (≥ 2 points change on each scale). Twenty-seven percent (*n* = 12) patients scored better and 16% (*n* = 7) worse on HADS-A, 24% (*n* = 11) vs. 22% (*n* = 10) on HADS-D, and 34% (*n* = 16) vs. 20% (*n* = 9) on DT scores. There was no statistically significant correlation between changes in DT, HADS-A, and HADS-D scores in the study population. We then classified CM patients into those who were diagnosed with meningioma within 12 months (*n* = 9) vs. longer (*n* = 22). OM patients were accordingly classified into operated within 12 months (*n* = 13) vs. longer (*n* = 17). There were no significant differences in mean HADS-A, HADS-D, and DT scores between those patient groups.

We performed a univariate logistic regression analysis to find the risk factors associated with increased anxiety (HADS-A), depressive (HADS-D) symptom severity, and distress (DT) scores at 3-month evaluation (Table 3). Tumor size was inversely associated with increased anxiety scores (*OR* 0.9 (0.9–0.98)). Tumor progression was associated with increased HADS-D scores (*OR* 6.3 (1.1–36.7)), and significant fatigue was inversely associated with DT score *OR* 0.1 (0.03–0.6). The association of HADS-A score with treatment group was close to statistical significance (3.4 (0.98–11.6), *p* = 0.054).

**Table 3:** Evaluation for possible risk factors for anxiety, depressive symptoms and distress at 3 months

Factors	HADS-A *OR (*95% CI)	HADS-D *OR* (95% CI)	DT ≥ 6 *OR* (95% CI)
Gender (male vs. female)	3.8 (0.7–21.0)	0.2 (0.0–1.1)	1.6 (0.4–7.3)
Age (≥ 65 years vs. younger)	0.6 (0.2–2.0))	1.1 (0.3–3.6)	3.5 (0.9–14.0)
Family status (single vs. partner/family)	0.8 (0.2–2.7)	0.7 (0.2–2.5)	1.1 (0.3–3.9)
Employment (full time vs. retired)	2.1 (0.5–8.4)	1.0 (0.3–3.8)	4.1 (0.7–22.6)
Education (higher vs. other/no)	1.2 (0.7–21.0)	-	-
ECOG (1 vs.0)	0.7 (0.1–3.5)	0.6 (0.1–2.6)	1.7 (0.3–8.8)
NANO score	1.0 (0.5–2.0)	0.7 (0.4–1.3)	0.8 (0.4–1.6)
Significant fatigue	0.8 (0.2–2.6)	2.4 (0.7–8.1)	0.1 (0.03–0.6)*
Wait-and-watch vs. operative treatment	3.4 (0.98–11.6)	0.8 (0.2–2.6)	0.6 (0.2–2.2)
Tumor location (convexity/falx vs. scull base)	1.0 (0.3–3.5)	0.7 (0.2–2.7)	2.9 (0.8–11.2)
Tumor size (mm)	0.9 (0.9–0.98)*	1.0 (0.9–1.0)	1.0 (1.0–1.0)
Time since diagnosis/operation (months)	1.0 (0.9–1.0)	1.0 (1.0–1.0)	1.0 (1.0–1.0)
Tumor progress	0.4 (0.1–2.3)	6.3 (1.1–36.7)*	0.7 (0.1–5.3)

*asterisk marks statistically significant values

### Course of recruitment

The recruitment of patients to the study started in 2018 and ended in 2020, which meant that some of the patients’ responses could have been influenced by lockdown measures, implemented in Germany due to COVID-19 pandemic. There were 11% (7 out of 62) patients in total who were recruited after implementation of lockdown in Germany on 22nd March 2020, 6 of them participated in the 3-month survey. Seventeen percent (8 out of 47) patients responded to 3-month survey after the start of lockdown. There was no significant difference in mean HADS and DT scores between patients recruited before and after the lockdown as well as no difference between the groups.

## Discussion

In this study, we found that the level of distress in patients with CM and OM meningiomas was high at the time of out-patient visit and remains high at home. It was not associated with the time since diagnosis or operation.

Many psychosocial factors might influence the level of distress and HRQoL of meningioma patients. The patients suffer from limitations of cognitive, emotional, and social function [21]. The psychological distress might be caused by even incidental radiological findings [6]. Moreover, follow-up might be distress-provoking. Scan-associated distress is a known phenomenon in tumor patients, causing some sort if psychological distress in majority of patients [1], although it might bring alleviation in some cases [26]. We have found that the level of distress at the follow-up visit in the same patient population as in current study was very high: the number of patients with increased values in HADS-A score is over 40% and HADS-D score over 70% at the time of the out-patient visit [12]. Contrary to expectations that the high number of distressed patients might be associated with a follow-up visit, scan-associated distress, or fear of tumor growth or relapse in this imaging, we did not find significant reduction of distress after 3 months. Moreover, the number of patients with normal values remained under 10% in both HADS scales. The underlying cause of high level of distress might be associated with a fear of tumor recurrence or progression [16]. The number of patients with meningioma having such fear is comparable to other brain tumors, such as gliomas [13], with significantly different prognosis. Even though the data on distress variation over time is limited, no changes in stress level were found in glioma patients during a 3-month follow-up [8]. A previous study in our clinic conducted on a different patient population demonstrated similar proportions of DT ≥ 6 in patients with high grade glioma (41%) and meningioma (39%) [24]. As only patients with good performance and neurological status were recruited in current study, it suggests that psychological factors have a paramount role for HRQoL in patients with meningioma.

There might be an association between diagnosis of meningioma and psychiatric disorders. In general, depression prevalence among brain tumor patients is between 10 and 40% [19]. Over 10% of patients with untreated meningioma are diagnosed with a mental health disorder within a year [19]. Moreover, general use of antidepressants (ADs) was an independent predictor of meningioma recurrence [15]. However, the increased use of antidepressant drugs in meningioma patients could be traced back longer than the median waiting time for the surgery, indicating that the patients with depressive symptoms might be more likely to receive cranial imaging and eventually be diagnosed with asymptomatic tumors [27]. Depression might be a presenting sign of meningioma and its prevalence possibly increased with an anterior location of the tumor [14]. How the level of anxiety and depression develop after the meningioma resection is not clear. For example, there was a decrease of mental distress and anxiety after the operation, no change in depression score was found [31]. The use of antidepressants was higher before meningioma surgery and continued to increase afterwards; interestingly, the use of sedatives was comparable to the normal population before the surgery, peaked at the time of operation, and remained increased afterwards [27]. Another study reported a significant reduction in mean depression scores after surgery, but not in anxiety scores [34]. In our study, the number of patients with an increased anxiety and depressive symptom severity was highly independent from their management strategy. Moreover, we found a significant association between higher HADS-D scores at t2 and tumor progress in a regression analysis, indicating that “bad news” during the follow-up can contribute to distress at home setting.

The prevalence of increased anxiety and depressive symptom severity according to HADS score in our study was higher than in most other studies [5, 25, 28], which might be due to regional differences or selection bias. Other factors that contribute to the high levels of depression and anxiety in this population must be assessed as well. For example, limitations and fears due to COVID-19 pandemic may cause significant stress for patients with brain tumors as well [30]. Even though the subgroup of patients in this study that were investigated after the start of lockdown was small, no considerable difference between responses were noticeable. According to a retrospective cohort of patients diagnosed with an incidental intracranial meningioma, approximately 10% underwent treatment within 8 years, and in a third of these patients, the indication was solely patient preference [11]. In our study, the level of distress was similar between patients that were diagnosed or operated on meningioma within a year vs. patients who were followed up for a longer period of time. This finding suggests that meningioma-associated distress persists for a long time. In certain cases, resection of an asymptomatic tumor might not bring a psychological relief the patient is seeking. Therefore, a psychooncological help might be necessary even for those patients who are followed-up for many years.

The cooperation between surgeons, neurooncologists, and psychooncologists, the development of supportive sources for the postoperative patients, and the patients with incidental meningioma might help reduce distress and improve their quality of life.

### Limitations

There are several limitations of this study that need to be considered. A small sample size, patient drop-out for t2 assessment, and recruitment in a tertiary care center limit the generalizability of the data. Secondly, to limit the influence of neurological deficits and poor performance on HRQoL, the study included only patients with good functional status. No psychological interview was done to validate the psychological burden assessed by the questionnaires.

## Conclusion

Psychological distress in conservatively managed accidental meningiomas and postoperative meningiomas with excellent outcome is high. The level of stress is not associated with an out-patient visit and remains high at home. Further investigations are needed to identify and counsel the patients at risk.

## Data Availability

The data presented in this study are available on request from the corresponding author.
